# Immune Response Modulation by Caliciviruses

**DOI:** 10.3389/fimmu.2019.02334

**Published:** 2019-10-01

**Authors:** Yoatzin Peñaflor-Téllez, Adrian Trujillo-Uscanga, Jesús Alejandro Escobar-Almazán, Ana Lorena Gutiérrez-Escolano

**Affiliations:** Departamento de Infectómica y Patogénesis Molecular, Centro de Investigación y de Estudios Avanzados, IPN, Mexico City, Mexico

**Keywords:** calicivirus, apoptosis, immunopathogenesis, replicative complexes, HuNoV, FCV, RHDV, MNV

## Abstract

Noroviruses and Sapoviruses, classified in the *Caliciviridae* family, are small positive-stranded RNA viruses, considered nowadays the leading cause of acute gastroenteritis globally in both children and adults. Although most noroviruses have been associated with gastrointestinal disease in humans, almost 50 years after its discovery, there is still a lack of comprehensive evidence regarding its biology and pathogenesis mainly because they can be neither conveniently grown in cultured cells nor propagated in animal models. However, other members of this family such as Feline calicivirus (FCV), Murine norovirus (MNV), Rabbit hemorrhagic disease virus (RHDV), and Porcine sapovirus (PS), from which there are accessible propagation systems, have been useful to study the calicivirus replication strategies. Using cell cultures and animal models, many of the functions of the viral proteins in the viral replication cycles have been well-characterized. Moreover, evidence of the role of viral proteins from different members of the family in the establishment of infection has been generated and the mechanism of their immunopathogenesis begins to be understood. In this review, we discuss different aspects of how caliciviruses are implicated in membrane rearrangements, apoptosis, and evasion of the immune responses, highlighting some of the pathogenic mechanisms triggered by different members of the *Caliciviridae* family.

## Introduction

Human caliciviruses (HuCVs), comprised by human noroviruses (HuNoV) and sapoviruses (HuSaV), are the leading cause of acute gastroenteritis globally (1), affecting millions of people each year (1, 2). They are considered the most common cause of acute diarrhea in both children and adults in industrialized countries; however, almost 50 years after its discovery, there is still a lack of comprehensive epidemiological evidence of the role of noroviruses in developing countries (3). Although most noroviruses have been associated with gastrointestinal disease in humans, knowledge regarding its biology and pathogenesis have been hampered because they cannot be conveniently grown in cell culture or propagated in animal models (4, 5). As a result, much of our knowledge on the basic mechanisms of norovirus biology has been largely inferred from studies on other animal caliciviruses that can be successfully propagated *in vitro* as well as in live models (5).

The *Caliciviridae* family is composed of small non-enveloped viruses of ~27–35 nm in diameter, with a single-stranded RNA of positive polarity genome of ~7.6–8.6 kilobases (kb) in length. This family currently comprises five genera: *Norovirus, Sapovirus, Lagovirus, Vesivirus, and Nebovirus* that are ubiquitous in the environment. Moreover, other six new genera: *Recovirus, Valovirus, Nacovirus, Bavovirus, Minovirus, and Salovirus* have been proposed (6–12).

Caliciviruses have a broad host range and cause a wide spectrum of diseases in their hosts, including digestive tract infections, vesicular lesions and reproductive failure, stomatitis, upper respiratory tract and systemic diseases, and hemorrhagic disease (6).

### Replicative Cycle

#### Calicivirus Attachment, Entry, and RNA Uncoating

Caliciviruses require attachment to their target cells through the interaction with oligosaccharides present in the cell surface. Many noroviruses, neboviruses, and lagoviruses require saccharides in histo-blood group antigens (HBGAs) [reviewed in (13) and (14), whereas vesiviruses and murine norovirus (MNV) use sialic acid (15, 16)]. Bile salts and divalent cations are also key mediators of norovirus entry (17). Like many other viruses, the members of the *Caliciviridae* family enter their host cell by receptor-mediated endocytosis. Protein receptors of three caliciviruses have been identified; the Junctional Adhesion Molecule 1 (JAM-1) for FCV (18, 19) and for Hom-1 calicivirus on human cells (20), and the CD300lf and CD300ld molecules for MNV (21, 22). Moreover, Annexin A2 has been implicated in the attachment and entry of the Rabbit vesivirus (RaV) (23). Although no more proteinaceous receptors have been described for other caliciviruses, it is known that RHDV interaction with HBGAs triggers the entry to its target cells (13); moreover, occludin is required as a coreceptor for porcine sapovirus (PSaV) entry to epithelial cells from porcine kidney LLC-PK cells with dissociated tight junctions (24).

Caliciviruses enter their host cells by triggering different endocytosis pathways following receptor engagement (24–27). The acidification of late endosomes is an essential step in the viral entry process (28, 29); however, the ways by which the virus escapes the endosome degradation and delivers the genome into the cytosol are poorly understood. New insights into calicivirus uncoating process come from a recent finding that demonstrates the formation of a portal on the capsid of FCV after JAM-1 engagement that allows the opening of the capsid shell for the release of the genomic RNA into the cytoplasm passing through the endosomal membrane (30).

## Calicivirus Genome Translation and Replication

**Figure 1:**
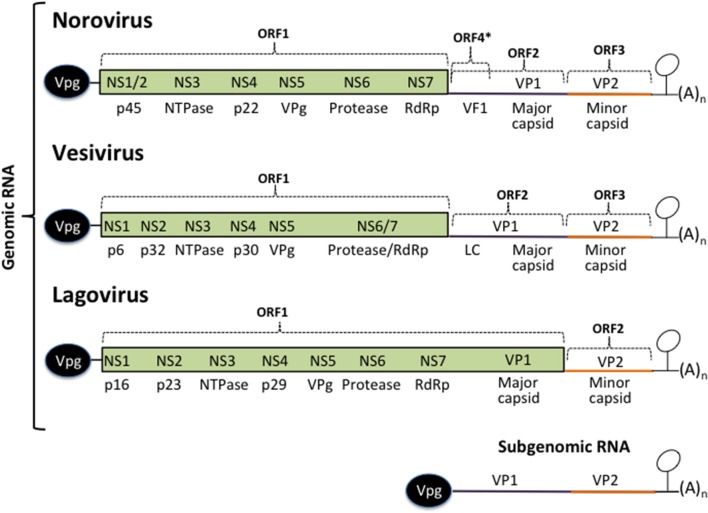
Calicivirus genome organization. All calicivirus genomes are 5′ Vpg-linked and polyadenylated at their 3′ ends. They contain an ORF 1 that encodes for the non-structural proteins NS1–NS7. The structural proteins VP1 and VP2 are encoded form the ORF 2 and 3 (*Noroviruses* and *Vesiviruses*) or form the ORF 1 and 2 (*Lagoviruses, Neboviruses, Becoviruses*, and *Sapoviruses*) are translated form the subgenomic RNA. The MNV Virulence Factor 1 (VF1) encoded from the ORF 4, the FCV Leader of the capsid (LC), and HuSaPV ORF-3 protein are unique among caliciviruses. Stem-loop structures present in the 3′ UTRs form the genomic and the subgenomic RNAs are indicated.

Calicivirus genome translation occurs immediately after the viral genome is released into the cytoplasm. Its genome is single-stranded of positive polarity RNA that is covalently bound to the viral protein genome-linked (VPg) at its 5′ end, and is polyadenylated; it is flanked by two short non-translated regions (NTR) that contain important regulatory elements for viral translation, replication, and morphogenesis (Figure 1). VPg linked to the 5′ end of the calicivirus genome serves as a proteinaceous cap that interacts with translation initiation factors, including the canonic cap-binding proteins; eIF4F, eIF4E, eIF4A, and eIF3 (31–38).

Calicivirus genomes are of ~7.5–8.6 Kb in length and contain from two to four open reading frames (ORF). The non-structural (NS) proteins are encoded in the ORF1, expressed as a polyprotein that is cleaved by the action of its own protease into 6 or 7 individual proteins designated non structural (NS) 1 through 7: NS1/2 or N-term, NS3 or helicase, NS4, NS5 or VPg, NS6 or protease, and NS7 or RNA dependent RNA polymerase (RdRp). The structural proteins VP1 and VP2 are encoded in the ORF2 and 3 from the subgenomic RNA as in noroviruses and vesiviruses; other caliciviruses encoded VP1 in the ORF1 [reviewed in (39, 40)]. MNV contain a fourth ORF that encodes the virulence factor (VF) 1, involved in evasion of innate immunity (41). In FCV, VP1 is encoded as a precursor that is cleaved by the viral protease-polymerase NS6/7 to produce the mature VP1 and a small protein of 124 amino acids known as the leader of the capsid protein (LC) (42) which has recently been shown to be involved in triggering apoptosis (43). The NS proteins, enhance translation rounds of the viral genome and lead the cell to the establishment of a pathogenic phenotype, inducing membrane proliferation that will induce the formation of the replication complexes (RCs), or viral factories and alter the vesicular secretory pathway to evade both the innate and acquired antiviral immune response and trigger apoptosis.

Calicivirus genome replication occurs in the RCs and involves the production of a negative-sense RNA template through the action of the NS7 protein, from which the genomic and subgenomic RNAs are produced (4, 39). Both VP1 and VP2 translation also takes place near the RC at late stages of infection.

### Maturation and Exit

Calicivirus exit has been associated with the induction of apoptosis as a mechanism to facilitate the dissemination of viral progeny in the host. Moreover, Santiana et al. have reported a non-lytically release of norovirus particles inside exosomes into stool that contribute to the fecal-oral transmission of multiple viral particles collectively to the next host (44). The role of apoptosis will be described in more detail below since it is essential for the establishment of the calicivirus immunopathogenic phenotype.

## Cytopathic Effect and Membrane Rearrangements in Calicivirus Infected Cells

All positive-stranded RNA viruses replicate in association with cytoplasmic membranes from the host cell where membrane compartments, known as RCs or viral factories are induced by viral NS proteins in coordination with host factors to facilitate productive RNA replication. Packaging of the viral genome also occurs in these compartments, providing an environment protected from host cell immunity. The membrane rearrangements induced by these viruses can be derived from a variety of organelles including the endoplasmic reticulum (ER) and Golgi apparatus, lipid droplets, endosomes, lysosomes, peroxisomes, chloroplasts, and mitochondria [reviewed in (45, 46)].

The first reports regarding the alteration of intracellular membranes caused by a calicivirus infection were published since the early 70s (47–49). In FCV, proteins p32 (NS2), p39 (NS3), and p30 (NS4) proteins were found to cause ER-derived origin membranous vesicles (50). Thus, as suggested by Bailey et al. the initiation step of RC formation may take place on the ER, but other components of the secretory pathway may be involved as this structure matures (50).

The expression of the RHDV viral protein p23, which is equivalent to the MNV NS1/2 and homolog of the FCV p32, has been shown to localize in the ER, as well as the helicase p29 (51). Interestingly, expression of the recombinant RdRp alone or in the context of the entire polyprotein from both RHDV and RCV cause a striking redistribution of Golgi but not ER membranes (51–53).

The expression of the N-terminal (NS1/2) non-structural protein from NV, was found to colocalize with the Golgi apparatus and induce intracellular membrane rearrangements (54), in contrast to the ER localization patterns of the homologous proteins from RHDV, MNV, and FCV (51, 55). A phenotypically abnormal Golgi was also shown as the result of both NV replication and the expression of the NS protein p22 (NS4) that was also involved in the inhibition of the cellular protein secretion pathway (ER to Golgi trafficking; 57). The antagonism of the secretory pathway produced by NV NS4 has been proposed to facilitate viral pathogenesis, probably as an interferon (IFN) and/or cytokine signaling deactivator (56). More recently, the expression of norovirus GII.4 ORF1 polyprotein was shown to produce the accumulation of single, double, and multi membrane vesicles likely built from the ER, that resemble those reported during MNV infection (57). Moreover, the expression of single proteins revealed different membrane alterations: NS1/2 induced proliferation of smooth ER membranes, NS3 was found associated with ER membranes around lipid droplets, and NS4 caused an even more pronounced effect than NS3 and was the only protein capable of inducing both single and double-membrane vesicles when expressed alone (57).

The differences in the intracellular localization of the NS proteins from several members of the *Caliciviridae* family, in both transfected and infected environments, indicate a high level of variation in the intracellular replication mechanisms; however, as with many other positive RNA viruses, membranes of the secretory pathway participate in the RC formation. Therefore, besides its role in viral replication, membrane rearrangement, and fragmentation of the Golgi network may also result in the alteration of the intracellular transport and secretion of host cell proteins involved in the evasion of host immune responses.

## Apoptosis in Calicivirus Infected Cells

During calicivirus infection, intrinsic apoptosis occurs to facilitate the dissemination of viral progeny in the host (58–63). Recent evidence suggests that apoptosis is used by noroviruses as a mechanism to suppress the translation of induced interferon-stimulated genes (ISG) in order to diminish the host innate immune response to infection (64).

The first reports of apoptosis induced by a calicivirus were observed in experimentally infected rabbits with RHDV that undergo liver cell death due to apoptosis (59, 65). Moreover, all cell types supporting viral replication in experimentally infected rabbits present apoptosis upon infection, such as lung macrophages, intravascular monocytes, endothelial cells (59), granulocytes, and lymphocytes (66). The RHDV VP2 (VP10) was the first viral protein associated with the induction of apoptosis in transfected cells (67). More recently, the ectopic expression of the viral trypsin-like cysteine-protease NS6 was found to cause the activation of caspase−3,−8, and−9 in rabbit (RK13), as well as in human (HepG2 and HeLa) cells (68), suggesting that apoptosis plays a central role in the pathogenesis of this virus.

Intrinsic apoptosis in FCV infection has been well-characterized and several reports have evidenced both morphological (chromatin condensation, DNA fragmentation, plasma membrane blebbing, and cell shrinkage) as well as molecular changes (Bax translocation to the mitochondria, cytochrome C and the Second Mitochondria-derived Activator of Caspases, and Direct IAP-Binding protein with Low PI, Smac/DIABLO release to the cytosol, and caspase−9 and−3 activation) (43, 58, 61–63). The FCV protein LC, which is unique among the *Caliciviridae* members, is responsible for the cytopathic effect characterized in infected CrFK cells by cell rounding and caspases activation and induction of the intrinsic apoptotic pathway (43, 69).

MNV infection is known to cause the induction of mitochondrial apoptosis via activation of caspase−3,−7, and−9 as well as induction of cathepsin B activity (60, 70). Even though, no single MNV-1 protein has been associated with the downregulation of survivin or induction of apoptosis (60), the expression of the MNV ORF1 polyprotein induces apoptosis in a virus-free cell model characterized by caspase-9 activation and survivin downregulation (71), indicating that one or more NS proteins are pro-apoptotic in the absence of viral replication. Furthermore, caspase-mediated cleavage of MNV-CR6 strain NS1/2 protein potentiates apoptosis through further caspase activation (72).

Replication of HuNoV causes apoptosis in the epithelial cells of small intestinal tissue sections (duodenum) of pig enterocytes characterized by nuclear displacement, chromatin condensation, and reduced number of organelles (73). The same morphological changes as well as caspase-3 activation were observed in infected human epithelial cells (74), particularly in enterocytes and sub-epithelial macrophages from intestinal transplant recipients with calicivirus enteritis (75–77). The increased apoptosis shown in crypt enterocytes in these immunosuppressed patients is generally considered to be the cause of intestinal allograft rejection and enteritis, while increased apoptosis of villus enterocytes and sub-epithelial macrophages is associated with enteritis (75, 76).

*In vitro* studies regarding the viral molecules implicated in the induction of apoptosis have identified two different NS proteins form HuNoV GII namely NS4 protein (p20) from norovirus GII.4 variant 2002, and the helicase NS3 or NTPase (P41) from the norovirus GII.4/YJB1/2009/Chiayi. This pro-apoptotic activity was enhanced by the co-expression with N-term or p22 (78). However, no protein encoded by NV, a GI.I strain, has been implicated in the induction of apoptosis.

## The Immune Response to Calicivirus Infection

Calicivirus infection is generally acute, although it can also be persistent, lasting weeks or even months after the appearance of the first symptoms. Depending on the virus, the infection can be limited to certain organs or can be systemic. The immune response of calicivirus involves both the innate and acquired components; here, we want to briefly outline some general aspects of calicivirus immunity to better understand the strategies that these viruses have developed to evade the immune response.

### Innate Immune Response

The innate immune response triggered by calicivirus is mediated by the interferon (IFN) pathway, as has been reported in several virus families. During norovirus infection, potent antiviral activities induced by different types of IFNs have been documented. MNV infected cells increase the expression of IFN and IFN related genes (IRG) through the dsRNA PAMP receptor MDA-5 that recognizes the viral genome (79, 80). MDA5 activates the interferon response factors (IRF) 3 and 7 and the NF-κB pathways through the mitochondrial antiviral signaling (MAVS) protein, leading to the expression of the interferon stimulated genes (ISGs) and pro-inflammatory cytokines. HuNoV RNA replication is also sensitive to several types of interferon, and various ISGs such as RTP4, HPSE, RIG-I, and MDA5 were identified as anti-norovirus effectors; particularly, the dsRNA sensors MDA-5 and RIG-I are potent inhibitors of HuNoV (81). IRF activation also occurs during RHDV infection of rabbit peripheral blood cells, and the potential role of leukocytes and their cytokines in infection has also been studied (82, 83). Regarding FCV infection, the 2,280 strain activates the IFN-β promoter and expression (84), as well as other ISGs like IRF-1 that hinder viral replication (85).

### Adaptative Immune Response

The adaptive or acquired immune response in calicivirus infection involves both humoral and cellular elements that help to control and eradicate the infection. Specific antibodies have been detected in several animals and humans after being exposed to caliciviruses. Induction of a humoral response has been documented with an attenuated strain of FCV in housecats (86), and with a wild type strain of RHDV in young rabbits (87), although different levels of protection against reinfection are achieved; moreover, it is also known that humoral response is required to completely clear MNV infection (88). A high prevalence of serum antibodies to HuNoV has been widely documented in children and adults; however, the protective role in the infection clearance is still a subject of discussion (89). The production and characterization of monoclonal antibodies (mAbs) that recognize specific sequences or structural regions of the epitopes of HuNoVs viral-like particles (VLPs) have helped to identify and characterize the antigens in distinct strains of HuNoVs, as well as its prevalence in distinct HuNoVs genotypes and the capacity of the virus to overcome its blockage. Two extensive and complete reviews about the characterization of the antigen epitopes of the HuNoV genotype GII.4 and its use in providing a road map for the design of candidate vaccines to cover distinct strains with major prevalence around the world as well as in the antigenic diversity of noroviruses have been recently published (90, 91).

Being the intestinal mucosa the main niche of the norovirus infection, it is natural that an important element of the host's humoral response is the production of immunoglobulin A (IgA). It has been proposed that IgA confers certain degree of protection, since HuNoV-exposed patients that already had higher saliva-HuNoV specific IgA did not show gastroenteritis symptoms (92). Moreover, a higher preexisting fecal HuNoV-specific IgA inverse-correlated with both peak of virus levels in stool and time of virus shedding, suggesting that IgA not only confers protection, but can control and produce a quicker clearance of infection (92). Specific IgA sera from HuNoV GI.1-infected patients has the ability to neutralize GI.1 VLPs binding and other GI VLPs such as GI.3 and GI.4 to carbohydrate ligands, reinforcing the idea of some level of cross-reacting protection, at least in the same genogroup (93). Moreover, it seems like IgA is more effective than IgG in blocking the virion binding to HBGA (94).

Although not extensively known, a specific cellular immune response occurs during MNV [reviewed in (95)] and FCV infection, where both monocytes and monocyte-derived dendritic cells (DC) presenting FCV peptides are capable of activating T CD8^+^ and CD8^−^ in an *in vitro* model; moreover, FCV-specific CD4+ T cells are present in the spleen of vaccinated cats (95–97). Although more information regarding the role of T cells in controlling HuNoV infection is required, some information has been discussed previously (98, 99).

## Evasion of the Immune Response

Upon a viral infection, the host cells activate a complex immune response to eliminate the invading pathogens; however, viruses have developed different strategies to escape such immune control mechanisms and to complete an effective replication and spread to a new host [reviewed in (100–102)]. Caliciviruses, as positive-sense single-stranded RNA viruses that replicate in the cytoplasm, have developed different strategies to prevent recognition of its components.

### Evasion of Type I IFN Immune Response

IFN plays a role in controlling calicivirus infection, as expected these viruses have adopted mechanisms for evading IFN signaling. STAT-1 and IFNs type I and II have essential roles in the clearance of MNV infections since they limit the viral replication in the intestine and viral dissemination to peripheral tissues and prevent apoptosis in intestinal cells (103–105). However, infection with the persistent MNV strain S99 caused a poor STAT-1 activation and IFN-β production, suggesting that the establishment of persistent infection in mice is possibly the result of a hampered innate immune response.

Infection with the FCV strains F9, Bolin, HRB-SS failed to induce an IFN-β response (84). To this regard, Yumiketa et al. (106) reported that the expression of the FCV NS protein 39 (NS3) suppresses IFN-β and ISG15 mRNA production and IRF-3 phosphorylation and dimerization induced by dsRNA, suggesting that this viral protein hampers type 1 IFN production by preventing IRF-3 activation (106). The identification of the viral molecules and the mechanisms followed by these viruses to control the IFN response is an area of active research and the proteins involved in inhibiting the cellular protein secretion pathway are targets for investigation. One example is the HuNoV NS protein 22 that antagonizes the secretory pathway; thus, it is probably a key factor in deactivating IFN and cytokine signaling (56).

### Role of Type III IFN in Calicivirus Infection Tropism, Persistence, and Transmission

IFN pathway impairment may play a role in calicivirus persistence, as both IFN-β and IFN-λ levels are not augmented in mice infected with the MNV-CR6 persistent strain, in comparison to the non-persistent strain MNV-CW3 (107). Moreover, IFN-λ treatment in mice confers protection against MNV-CR6 persistent strain for up to 2 weeks, and mice receiving this treatment 25 days post infection with this strain, showed a reduction in viral shedding at 2 days of treatment and total virus clearance within 1 week (107), suggesting that the main site of action of IFN-λ is in the intestine, reducing viral dissemination. Another possible role of IFN-λ against MNV infection is protection from contagion; blood and intestinal overexpression of IFN-λ and its stimulated genes in IFN-α/β and IFN-γ knockout mice generated protection against MNV when exposed to seeders and infected mice shedding virus through their stool (108). The relationship between the gut microbiome and IFN-λ in calicivirus infection has been briefly studied using antibiotic-treated mice, where persistent infection and replication in the intestine is only prevented in treated mice that have a functional IFN-λ pathway. This review showed that the commensal microbiome in mice gut fosters MNV infection by downregulating the IFN-λ pathway (109).

### Interactions of Caliciviruses With the Microbiota

Many caliciviruses access their hosts through mucosal surfaces that contain a diversity of commensal pathogens; thus, in these sites they interact with hundreds of differential commensal bacteria, which are part of the host immune defense [reviewed in (109–111)]. Particularly, pathogens infecting the intestine, such as HuNoVs and MNVs encounter these microbes with a harmful of beneficial result to the host. On one hand, it has been reported that the *Lactobacillus* genus can inhibit MNV replication *in vitro* by the up-regulation of IFN-β and IFN-γ expression (112), indicating its protective role from this viral infection and suggesting that commensal bacteria in mucosal sites are part of the antiviral response against pathogenic viruses (110).

On the other hand, and contrary to this benefit of the gut microbiota, enteric bacteria are a stimulatory factor for norovirus infection; both HuNoVs and MNV require the presence of HBGA-expressing enteric bacteria to infect B cells *in vitro* and likely *in vivo* (113). More recently, the ability of different HuNoV strains to bind to naturally occurring bacteria strains isolated from human stool as well as to selected reference strains was reported (114). In this same work, a selective binding of Tulane virus, a calicivirus that infects the gastrointestinal tract of rhesus monkeys to some of these bacteria was also observed (114). Binding of caliciviruses to bacteria may facilitate the entry into their target cells and the development of infection; thus, microbiota could be a mechanism of calicivirus evasion.

### NS1 From Persistent MNV CR6 Strain Impairs IFN-λ Response

It has been reported that the NS1/2 gene of the persistent MNV-CR6 strain is responsible for the virus tropism and persistence, since a chimeric acute CW3 strain expressing the NS1/2 from CR6 becomes persistent, changing its tropism to the proximal colon of infected mice (72); however, only the NS1 region is responsible for this tropism and persistence. It was recently demonstrated that NS1 protein from MNV-CR6 persistent strain is a product of caspase 3 cleavage, and secreted by a non-classical secretion pathway, downregulating the IFN-λ response in the intestine of infected mice (115). Moreover, mice immunized with MNV-CR6 NS1 gained protection against infection and achieved a better prophylaxis than the one obtained by immunization with VLPs or VP1 protein alone. This phenomenon may be conserved between noroviruses, as the secretion of NS1 from HuNoV GI.1 has also been demonstrated. Thus, NS1 protein from MNV (and possibly from other caliciviruses) is an immune response modulator, particularly affecting the IFN III pathway and influencing virus tropism and host persistence.

### Calicivirus Infection and Translational Control

Viruses rely absolutely on the protein synthesis machinery of the host cells; thus, they have developed remarkable strategies to inhibit cellular protein synthesis to prevent competition of ribosome recruitment by host mRNAs. Moreover, inhibition of *de novo* cellular protein synthesis may also contribute to neutralize the stress responses and host innate defenses to infection (116).

Caliciviruses can alter the global translational control of the host by: (1) directly or indirectly targeting cellular translation factors and (2) by altering host ribonucleoprotein (mRNP) compartmentalization, particularly disrupting the assembly of cytoplasmic stress granules (SGs).

1) Targeting cellular translation factors. VPg from the FCV and MNV interact with eukaryotic initiation factors such as eIF3, eIF4E, eIF4A, and eIF4G to promote its own translation; thus hijacking the host protein machinery (31–34, 36). Moreover, infection with FCV alters the global host translation by targeting two eukaryotic initiation factors: eIF4G and the poly A binding protein (PABP) by the action of the viral protease-polymerase NS6/7 (117, 118). MNV infection causes phosphorylation of eIF4E through the MAPK pathway, contributing to changes in the translational state of specific host mRNAs (119). To this regard, a fraction of PABP is also cleaved during MNV infection by the action of the protease NS6 and as a consequence, a reduction in the translation of induced ISGs takes place (64).

Besides the direct targeting of cellular translation factors by viral proteins, induction of apoptosis by viral infections results in the activation of caspases, cellular proteases that also mediates the cleavage of cellular translation initiation factors (120). During MNV infection, cleavage of eIF4GI and eIF4GII occur as a consequence of caspase 3 activation; thus, apoptosis also contributes to the global inhibition of cell translation and evasion of the immune response (64). Caspases also causes the cleavage of nucleoporins (Nups), the principal components of the nuclear pore complex that mediate protein and RNA traficking between the nucleus and the cytoplasm (121). We have observed that during MNV and FCV infection, Nup270 (Tpr) and Nup153 are targets of caspases (unpublished data), suggesting that alterations in the nucleo-cytoplasmic transport may contribute to avert the innate immune response.

Thus, modification of translation initiation factors by calicivirus infection (summarized in Table 1), not only favors viral protein synthesis but also hinders the translation of genes induced by the innate immune response (64, 103).

**Figure 2:**
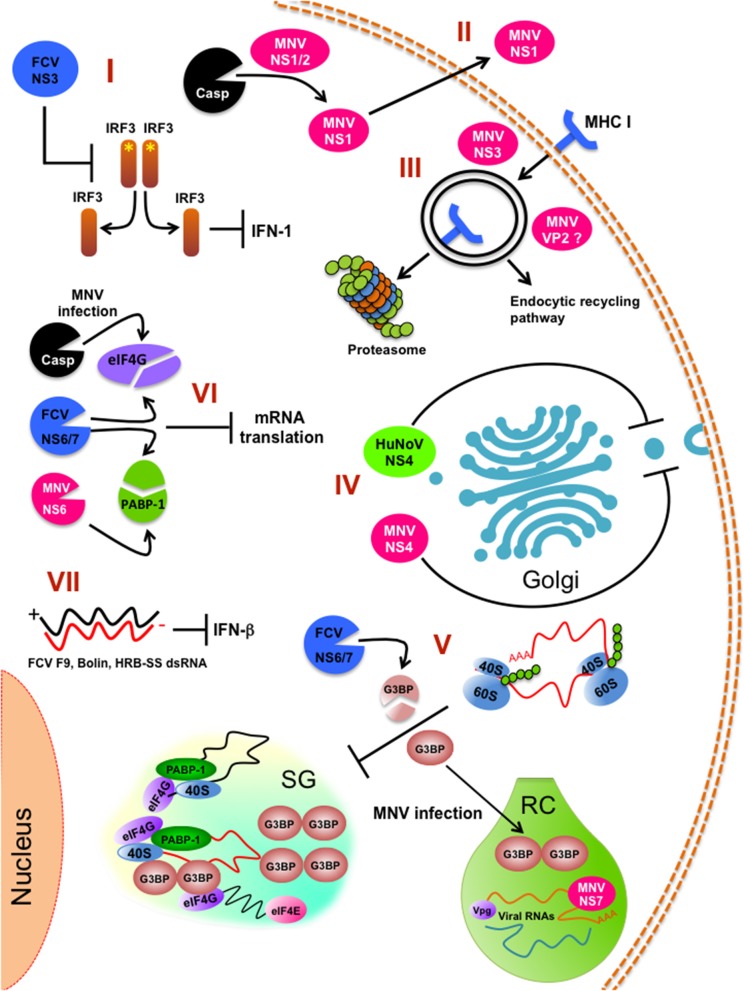
Innate immune pathways inhibition by calicivirus infection. (I) FCV NS3 prevents IRF-3 activation and hampers type 1 IFN production. (II) Caspase-mediate cleavage of MNV NS1/2 for persistent infection of intestinal epithelial cells. (III) Internalization and degradation of MHC I by the action of MNV NS3 protein. (IV) Inhibition of the cellular protein secretion pathway (ER to Golgi trafficking) by HuNoV NS4 (p22) and MNV NS4 (p18). (V) Stress granules (SG) impairment by FCV and MNV infection. (VI) Inhibition of the global cell translation by MNV and FCV infection. (VII) Impairment of INF-β response by FCV strains F9, Bolin, HRB-SS. RC (replicative complex) is indicated. ^*^Indicates phosphorylation.

2) Compartmentalization. Upon infection, inhibition of bulk host protein synthesis can also be regulated by the induction of stress granules (SGs) that are important components of the host antiviral defense. SGs are non-membranous, transiently assembled ribonucleoprotein (RNPs) complexes were cell translation can be stalled by sorting non-essential mRNA away in response to stress [reviewed in (124–126)]. However, as gene expression of heterologous viral RNAs can also be regulated in these structures, inhibition of SG formation can occur by viraly-encoded factors to confront this antiviral response and maximize virus replication efficiency. FCV infection impairs the assembly of SGs through the cleavage of the SG-nucleating proteins (G3BP1 and G3BP2) by the viral protease-polymerase NS6/7 (122), while MNV infection restricts SG nucleation and formation by recruiting G3BP1 protein to its replication sites, with the equally consequence to prevent SG formation and enhance replication (123) (Figure 2).

**Table 1:** Calicivirus proteins involved in translational control.

**Virus**	**Viral proteins**	**Cellular protein targets**	**Mechanisms**	**References**
FCV MNV	VPg	eIF3, eIF4F	Interacting with cellular factors and usurp the host protein synthesis machinery	(31, 33, 34, 36)
FCV	NS6/7 protease/polymerase	eIF4G	Processing translation factors and inhibiting global cellular protein synthesis	(118)
FCVMNV	NS6/7 and NS6 proteases	PABP	Reduction in the translation of induced interferon stimulated genes (ISGs)	(64, 117)
MNV		eIF4E phosphorylation	Affecting translational state of specific host mRNAs	(119)
FCV	NS6/7 protease/polymerase	G3BP1 and G3BP2	Impairs formation of stress granules	(122)
MNV		G3BP1	Impairs formation of stress granules	(123)

### High Mutational Rates, Genetic Diversity, and Evasion of the Immune Response

RNA viral populations consist on a spectrum of different genomes that result of the high mutational rates derived form the characteristically low fidelity of viral RdRp, as well as molecular recombination and gene segment reassortment. These viruses with extremely high mutation rates exhibit a faster replication, that results in the establishment of the infection in the cell before the immune response can hamper it, and present significant genetic diversity that allow them to evolve, and thus, avoid the cell antiviral systems (127).

One of the first studies on calicivirus mutation rate and evolution showed that isolates collected during a year from a single immunosuppressed patient with chronic diarrhea and viral shedding of HuNoV, presented few changes in the ORF1, but accumulated changes in the P domain of the capsid protein VP1, that were subject to immune pressure (128). Similar changes have been observed in the SaV VP1 codifying region (129). Mutations in the viral capsid protein are one of the immunity-driven mechanisms, to evade a humoral response of the host; particularly information encoded in the P2 subdomain could direct different mechanisms to escape immunological memory [reviewed in (130)].

Analysis of the fidelity of the replicase from HuNoVs involved in gastroenteritis outbreaks have shown that the RdRp from the HuNoV GII.4 strain, responsible for the majority of outbreaks worldwide, has the highest evolution rate compared with other less frequently detected strains, suggesting that the high prevalence of certain strains around the world is a consequence of the genetic diversity of their genomes, which result in a greater capacity to response to new environments. Interestingly, comparison of the evolution properties of several pandemic GII.4 strains to non-pandemic strains found that GII.4 viruses undergo evolution at a much higher rate than the non-pandemic strains (131). Moreover, the presence of non-synonymous mutations in all the HuNoV genotypes analyzed were localized to common structural residues in the capsid, which indicates that these sites are most probably under immune selection (131).

High mutation rate in calicivirus and its importance to achieve efficient viral replication can be observed when a strain with a detrimental phenotype is repeatedly passed through cellular culture until it reaches wild type characteristics. Examples of this has been documented, as the reversion of the LC mutants in FCV (69) and VF1 mutants in MNV (41). Studies in the evolution of an hypervariable region of the FCV capsid protein known to contain neutralization epitopes has shown that the altered viral antigenic profile produced in persistently infected cats generate sequences not detected by the host, that may also result in an evasion of the immune response (132).

### MNV VF1 Factor Antagonizes the Innate Immune Response

A rather unique characteristic in MNV's genome is the presence of a fourth ORF that encodes for the protein VF1 in the subgenomic RNA. The protein VF1 locates to the mitochondria and has been suggested to participate in the control of the virus-induced apoptosis and an anti-innate immune activity through the downregulation of the interferon immune response. The protein VF1 interferes with the expression of antiviral genes including IFN-β, the IFN-stimulated gene ISG54, and CXCL10 (41, 133, 134). A similar ORF that overlaps the VP1 coding region is present in genogroup I sapoviruses, although the production of a protein from this ORF has not been demonstrated (135); yet, its conservation in both MNV and sapoviruses suggests that the product of this ORF plays a critical role in virus pathogenesis.

### Prostaglandins and Nitric Oxide Production in Calicivirus Infection

Prostaglandins (PG) are IFN antagonists that modulate the production of the innate immunity effector nitric oxide (NO), involved in the control of many infections. It has been reported that levels of NO increase in the digestive tract of patients suffering from acute gastroenteritis caused by noroviruses (136). During PSaV infection, there is an increased activation of the cyclooxygenase-2/Prostaglandin E_2_ (COX-2/PGE_2_) and an inhibition of the NO production. The inhibition of the COX/PGE_2_ pathway caused an increase of NO production and a reduction of the viral replication, indicating that PSaV hampers the antiviral response to provide an environment appropriate for efficient replication. The increase of both COX-2 mRNA and protein levels is produced during the infection and by the expression of the viral proteins VPg and protease-polymerase NS6/7 (137). A similar activation is also observed during FCV and MNV infection, suggesting a crucial role for the COX2/PGE_2_ signaling pathway in the replication of the *Caliciviridae*; however, the viral factors that modulate the evasion of the immune response in these particular viruses remain to be determined (138).

### Impairment of Antigen Presentation by MNV

As previously discussed above, the mechanisms underlying norovirus persistence or clearance are not well-understood; however, studies of MNV persistence strains and its effect on lymphocyte TCD8+ activation suggest that the infection of macrophages and dendritic cells with MNV impairs the antigen presentation pathway by reducing the surface expression of MHC class I proteins early during infection (111). This reduction is the consequence of the MHC class I internalization via the endocytic recycling pathway and proteasome-dependent degradation. This phenotype is likely to be caused by the NS3 protein, the NTPase involved in viral genome replication (Figure 2). This reduction of MHC class I levels hinders the presentation of viral peptides, the activation of CD8^+^ T cells, and the initiation of the cellular immune response (111). This evasion of antigen presentation most certainly has a role in the clearance of the virus from the host, because it is known that the CD8+ T lymphocytes of mice infected with the persistent MNV-CR6 strain have a differential gene expression than those of the acute strain CW3-infected mice; this distinct gene expression alters the TCD8+ cells localization in the mouse organism, as well as its capacity to respond to activation by proliferation. The results suggest that there are at least two ways of how calicivirus dampers TCD8+ mediated immunity: (1) the reduction in the presentation of MNV antigens by enteric infected cells through internalization of the MHC-I proteins and (2) the distinct expression of differentiation clusters in TCD8+ activated cells presented with the MNV persistent strain antigens, resulting in a suboptimal activation and relocalization of these cells, creating an special niche in the intestine, with fewer TCD8+ lymphocytes where MNV replication can be efficiently achieved (139, 140). The role of the NS1/2 protein of the MNV persistent strain CR6 in the TCD8+ cell response modulation has not been thoroughly studied, but it is known that a MNV CR6 strain lacking a functional NS1/2 (CR6D121/131G) cannot efficiently replicate in wild type nor in *Rag*^−/−^ mice lacking B and T cell immune response; suggesting that NS1 does not affect acquired immune response and, therefore the TCD8+ differential activation by the persistent strain has to be through another unknown mechanism (115).

Another protein that is involved in the modulation of the immune response is the MNV minor capsid protein VP2 that can regulate antigen presentation (133, 134). Some calicivirus proteins involved in the modulation of the immune response are shown in Table 2.

**Table 2:** Calicivirus factors involved in the modulation of the immune response.

**Genes**	**Virus**	**Viral factors**	**Immune regulatory functions**	**References**
*Norovirus*	MNV	Virulence factor 1	Downregulation of the IFN immune response	(41)
		NS3	Reduction of the MHC-I protein in the cell surface Decrease in viral antigen presentation Altered CD8+ T cells response	(111)
		Unknown	NO reduction by COX_2_/PGE2 pathway activation	(138)
		NS1/2	Impairment of IFN III pathway	(115)
		VP2	Regulation of antigen presentation	(133, 134)
	HuNoV MNV	NS4(p22) NS4 (p18)	Impairment of INF and cytokine signaling	(56)
*Vesivirus*	FCV	dsRNA	Induction of IFN-b promoter by FCV strain 2280 Lack of IFN-b induction by FCV strains F9, Bolin ad HRB-SS	(84)
		NS3 (p39)	Downregulation of Interferon 1 pathway by preventing IRF-3 activation	(106)
		NS6/7	Sgs assembly disruption by G3BP-1 and G3BP-2 cleavage	(122)
		Unknown	NO reduction by COX_2_/PGE2 pathway activation	(138)
*Sapovirus*	PSaV	VPg and NS6/7	NO reduction by COX_2_/PGE2 pathway activation	(137)

## Evasion of the Humoral Response by Caliciviruses

It is well-known that calicivirus infection triggers a humoral response with an impact in the control of the infection, virus propagation or spread through the host. The calicivirus capsids are composed of 180 copies of the VP1 protein that contains 2 principal domains: (1) The amino terminal S (shell) domain proximal to the viral genome, and (2) the carboxi-terminal P (protruding) domain, that dimerizes to form protrusions on the capsid surface. The P domain, the most common target for antibodies in the host is divided in 2 subdomains, P1 that interacts with the S domain and P2 that is the protruding region of P that contain the binding sites for cellular receptors and neutralizing antibodies (141). Multiple efforts for a vaccine development that generates humoral protection against HuNoV have been made; however, the high mutation rate in amino acids from the distinct capsid epitopes combined with selective pressure from the host, damper cross reaction, and longlasting protection both after vaccination and infection; constant changes in genomic populations thorough the world every 5 years are another major problem in vaccine development (142).

The D epitope, adjacent to the P domain in the HuNoV VP1, responsible for the binding with the glycans from the HBGAs, can experience variations in its sequence that result in both a change in affinity for a specific HBGA group as well as an escape from the blockade antibodies. The plasticity of this region has an impact in the tropism of a particular HuNoV genotype as well as in the herd immunity of a population exposed to a particular strain (98, 143).

Another mechanisms of HuNoVs involved in the evasion of the humoral response is the “breathing” of the viral capsid; this phenomenon result of slight spatial rearrangements of the virion epitopes reducing their exposure to hosts antibodies without an effect in infectivity (141). Moreover, amino acid changes in the regions surrounding the conserved epitopes that are constant targets for antibody blockage are also a mechanism of evasion of this response, since a steric effect can result in a reduced antibody binding to the epitope. Posttranslational modifications of amino acids in the viral capsid have also an effect in the virion binding to its HBGA co-receptor (144); however, its effect in the antibodies binding to the viral capsid remains to be determined.

The structure of HuNoV, MNV, RHDV, and FCV virions determined by cryo-electron microscopy studies have shown differences in the size of the P-S linker domains that result in distinct dominant epitopes and antibody binding sites [reviewed in (141)]. Particularly in MNV there is a greater flexibility between these P-S domains that could help the virus evade the humoral immune response through diverse mechanisms, like the P-S linker rupture by hosts proteases that results in the release of the P domains as decoys to avoid antibody interaction with the virion; however, since these conformational states seems to be not conserved among all calicivirus, they may not be a common strategy for a successful virus replication and thrive. To this regard, cryo-electron microscopy studies have shown that FCV binding to its receptor occurs in its compressed state (145).

Conformational changes in the P epitope have effects on the neutralizing capability of antibodies to successfully bind to the virion, as a closed or open state in the P dimer expose or hide distinct regions of the epitopes that result in a less efficient virion neutralization response. All these mechanisms regarding changes in the structure of the virion as a way to avoid the humoral immune response have been recently described in more detail in the excellent review by Smith et al. (141).

## Conclusions

All viruses encode for viral factors that are key elements in the regulation of the viral replicative cycle, and as a consequence, in the establishment of infection. During calicivirus infection, several cellular pathways are altered to achieve a successful viral production and viral spread, as well as an effective evasion of the immune response. Infections produced by the members of the *Caliciviridae* family depend on similar pathways; however, the viral factors involved in its regulation are not always the same, which may lead to the diversity of viral tropism and immunopathogenesis. Moreover, the corresponding proteins have multiple functions, not all of which are currently well-understood; thus, knowing the multiple roles of these viral factors will impact on the development of new strategies for the control of calicivirus infection both in humans and animals.

## Author Contributions

YP-T, AT-U, JE-A, and AG-E collaborated equally in the bibliographical research and writing. AG-E coordinated and edited the manuscript.

### Conflict of Interest

The authors declare that the research was conducted in the absence of any commercial or financial relationships that could be construed as a potential conflict of interest.
